# Complications of Internal Continuous and Perforating External Osteotomy in Primary Rhinoplasty

**Published:** 2017-05

**Authors:** Sadrollah Motamed, Alireza Saberi, Feyzollah Niazi, Hojjat Molaei

**Affiliations:** Department of Plastic and Reconstructive Surgery, Shahid Beheshti University of Medical Sciences, Tehran, Iran

**Keywords:** Osteotomy, Rhinoplasty, Continuous, Perforating, Complication

## Abstract

**BACKGROUND:**

Osteotomy is one of the major steps in rhinoplasty. The aim of study was to compare edema and ecchymosis after external and internal lateral osteotomy in patients who underwent rhinoplasty.

**METHODS:**

Based on a prospective randomized clinical trial, 168 osteotomies were performed through an external route in a perforating fashion and internal route in a continuous fashion at right or left side respectively in any patient. Subjective scoring system was applied to evaluate edema and ecchymosis on 1^st^, 3^rd^, 7^th^, and 30^th^ days after surgery.

**RESULTS:**

Edema and ecchymosis were the same in both types of osteotomies.

**CONCLUSION:**

Regarding edema and ecchymosis, there was not any significant difference between external and internal osteotomies in rhinoplasty.

## INTRODUCTION

**Fig. 1:**
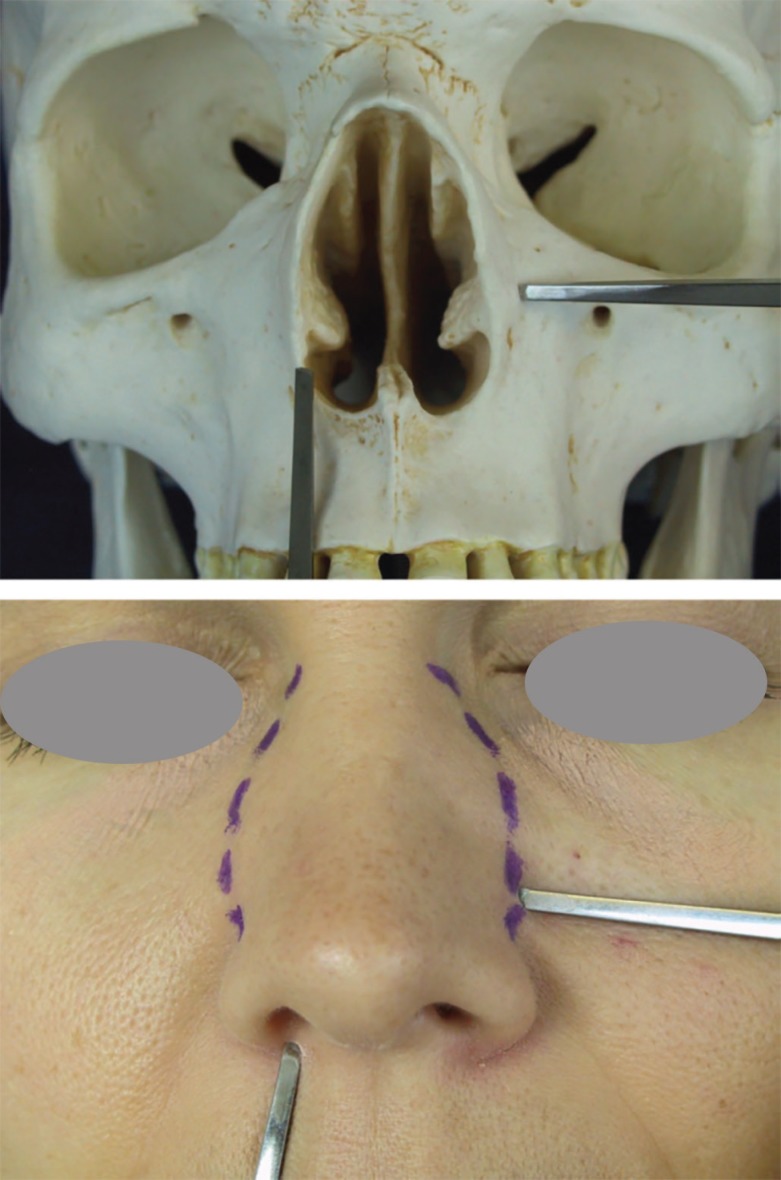
Internal vs external osteotomy

Nasal bone osteotomy as the most dangerous and less controllable stage of rhinoplasty, has its prominent role in ideal aesthetic results. Main indications of osteotomy include (i) Correction of open-roof deformity after dorsal hump reduction, (ii) Narrowing of nasal pyramid, (iii) Eliminating asymmetry or convexity and (iv) Straightening of convex nasal bones. Despite developments in instruments and techniques of osteotomy, there is not any unique accepted approach to deal and everybody relay on his taken rout.^1^^,^^2^ Lateral osteotomy (internally or externally) forces high energy on hard and soft tissues of nose and should be done without unwanted change and instability (Figure 1). 

Rees and Ford believed in external osteotomy because of decreased trauma to soft tissue, mucosa and peristeum.^3^^,^^4^ On the other hand, Tardy and Denneny confirmed internal osteotomy with 2-3 mm osteotome without protector and declared diminished edema, ecchymosis and mucosal destruction.^5^ Rohrich illustrated a low rate of tissue damage after external lateral osteotomy in a cadaveric study.^6^ Becker *et al. *showed edema and ecchymosis decrease after external osteotomy in their cadaveric samples.^7^ So, we decided to plan a study to evaluate different osteotomy techniques (externally vs internally) individually to overcome defects of previous studies by eliminating confounding factors and compare complications of these techniques.

## MATERIALS AND METHODS

Eighty four patients who were candidate for aesthetic rhinoplasty included in a prospective randomized study for 12 months. Patients completed consent form and divided randomly in two groups. Before operation, lab tests such as PT, PTT and CBC (complete blood count) were taken which all were in normal range and none of patients had any history of coagulopathy. Surgery was the same for both groups except for lateral osteotomy. Osteotomies were done by one surgeon who was expert in both techniques. Patients in the first group underwent external osteotomy with 2 mm straight osteotome without protector on the left side perforating and on the right side with continuous internal osteotomy and in the second group, these osteotomies were accomplished conversely.

All patients underwent general anesthesia. None of patients received corticosteroid injection. At first and 10 minutes before lateral osteotomy, mixture of epinephrine (1/100000) and lidocaine (1%) was infiltrated in medial and lateral osteotomy sites and lateral to frontal process of maxillary bone. Lateral osteotomy was done as the last step of operation. Periosteal elevation was accomplished in each technique. Before external lateral osteotomy, incising skin was conducted with 2 mm osteotome and interrupted osteotomy from periform aperture to intercanthal line and at last resulted into green stick facture with in-fracture finger push. 

In the same direction, internal osteotomy was performed with a 4 mm strait sharp osteotome on the opposite side of the patient. Termoplast was applied for 7 days and the patients received cephalexin capsule (500 mg) every 6 hours for 7 days and acetaminophen codeine (320 mg) for 24 hours. Edema and ecchymosis were measured by a person who was unaware of the kind of osteotomies at 1^st^, 3^rd^, 7^th^ and 30^th ^after operation and recorded. Findings were scored according to available samples of questionnaire. Our subjective scoring was based on Kara and Goylan study.^8^

**Fig. 2:**
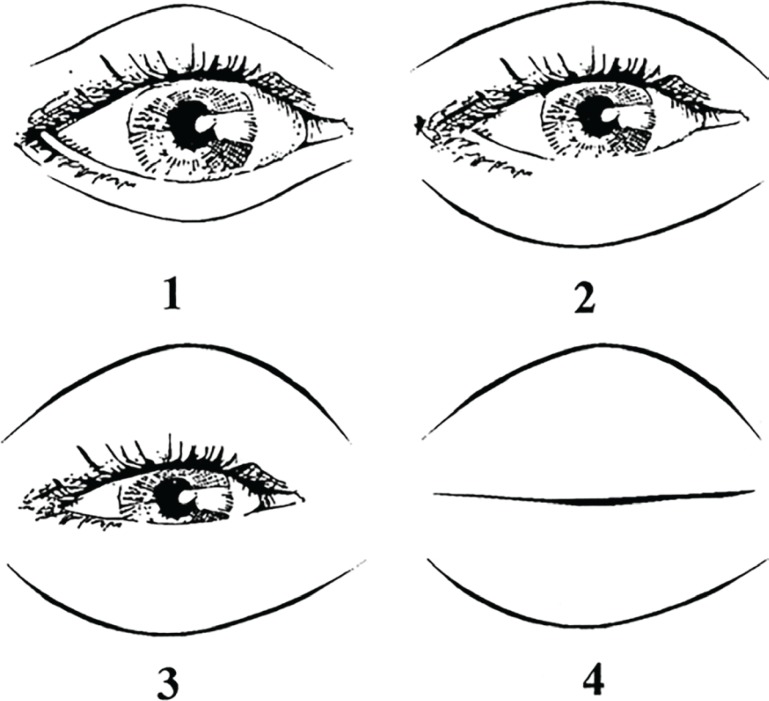
Scoring diagram for edema. Grade 1, no coverage of iris with eyelids. Grade 2, slight coverage of iris with swollen eyelids. Grade 3, full coverage of iris with swollen eyelids. Grade 4, full closure of eyes.^8^

**Fig. 3:**
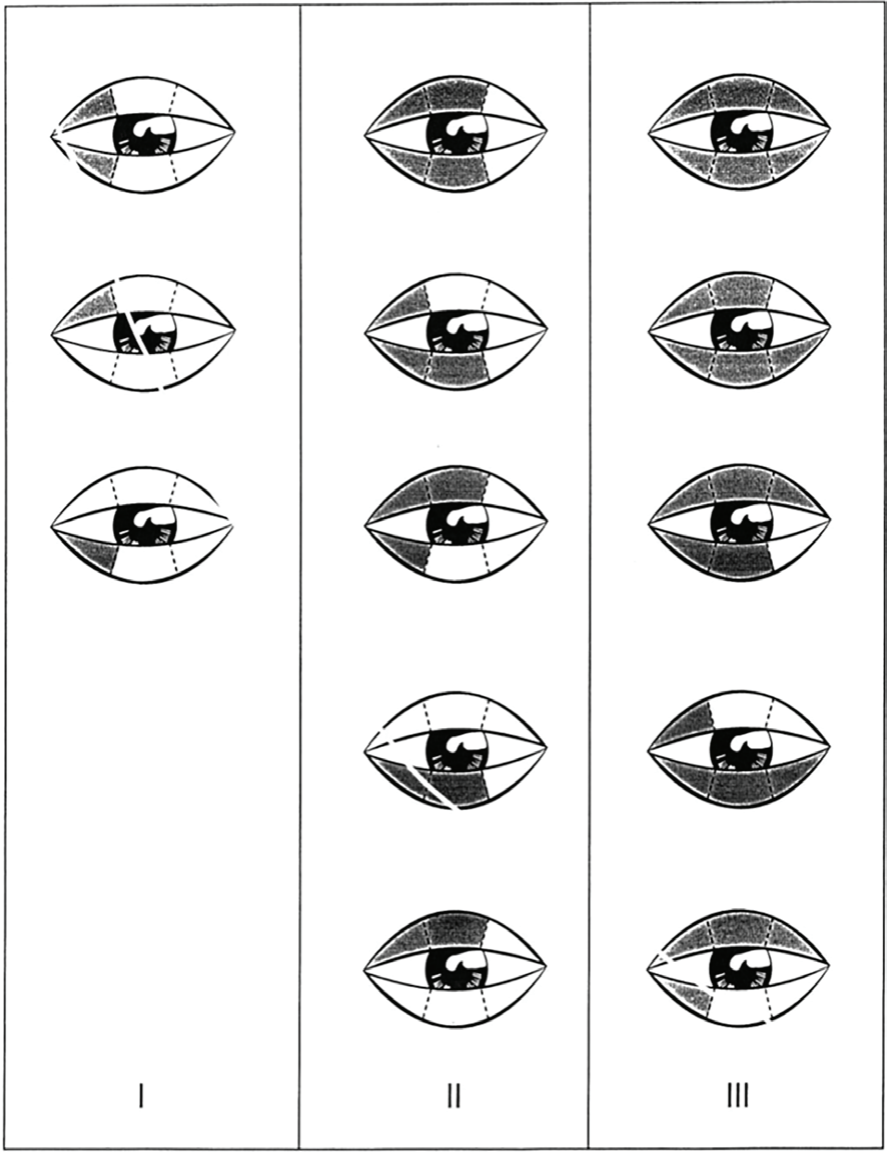
Scoring diagram for ecchymosis. Grade 1, ecchymosis up to the medial one-third part of the lower and/or upper eyelid. Grade 2, ecchymosis up to the medial twothirds part of the lower and/or upper eyelid. Grade 3, ecchymosis up to the full length of the lower and/or upper eyelid.^8^

Edema scoring was based on degree of coverage of iris and pupil with upper and lower eyelids: 1. No coverage of iris, 2. Little overage of iris with eyelids, 3. Complete coverage of iris with eyelids and 4. Complete eye closure (Figure 2). Ecchymosis scoring was based on amount of discoloration in bruised eyelids: 1. Limited to 1/3 medial upper/ lower eyelids, 2. Limited to 1/3 middle upper/ lower eyelids, 3. Limited to 1/3 lateral upper/lower eyelids. Generally, the highest score was recorded- without considering right or left side (Figure 3). Gathered data were entered in analytic software SPSS (Version16, Chicago, IL, USA) and analyzed with Mann Whitney and x^2^ tests. P value less than 0.05 was assumed significant.

## RESULTS

**Fig. 4:**
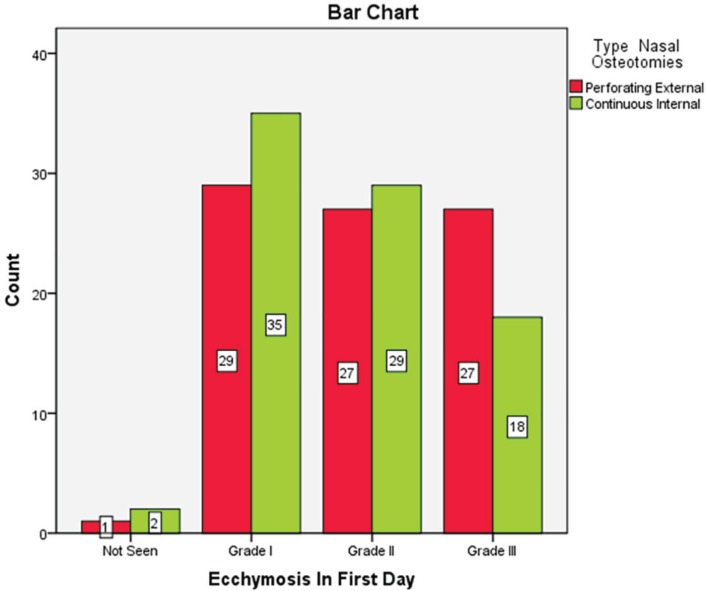
Ecchmosis in patients after one day of undergoing nasal osteotomy

**Fig. 5:**
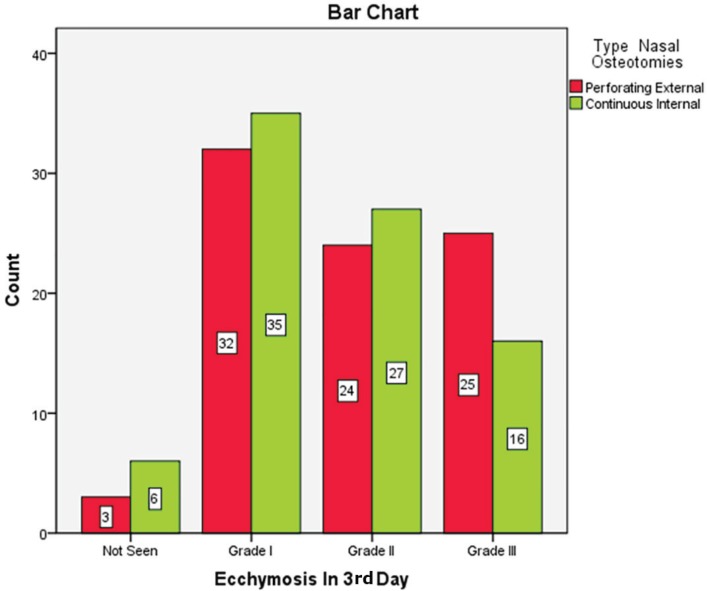
Ecchmosis in patients after 3 days of undergoing nasal osteotomy

Of 84 patients, 60 cases were female (71.4%) and 24 ones were male (28.6%). Seven patients underwent septoplasty (8.3%). There was not any patient with immunocompromised or coagulopathy problems. The most common ecchymosis in the 1^st^ day was grade 1. Twenty nine patients (34.5%) in external route and 35 cases (41.7%) in internal route had grade 1 ecchymosis on the 1^st^ day and there was no significant difference between the two methods (*p*=0.42) (Figure 4). The most common ecchymosis in the 3^rd^ day was grade 1. Thirty two patients (38.1%) in external technique and 35 cases (41.7%) in internal route had grade 1 ecchymosis on the 3^rd^ day and there was no significant difference between routes (*p*=0.34) (Figure 5).

**Fig. 6:**
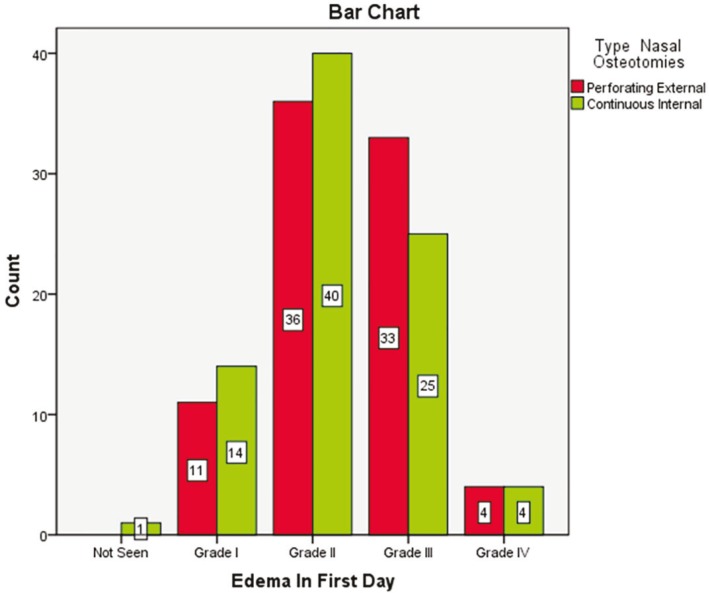
Edema in patients after one day of undergoing nasal osteotomy

Most of patients had no ecchymosis on 7^th^ day. Seventy three cases (86.9%) in external route and 74 cases (88.1%) in internal method lacked ecchymosis and there was no significant difference regarding types of osteotomy and ecchymosis (*p*=0.8). 98.8% patients did not have any ecchymosis on 30^th^ day with no significant difference (*p*=0.3). The most common edema rate in the 1^st^ day was grade 2. Thirty six patients (42.9%) in external route and 40 cases (47.6%) in internal way had grade 2 edema on the 1^st^ day and there was no significant difference between the two routes (*p*=0.6) (Figure 6).

**Fig. 7:**
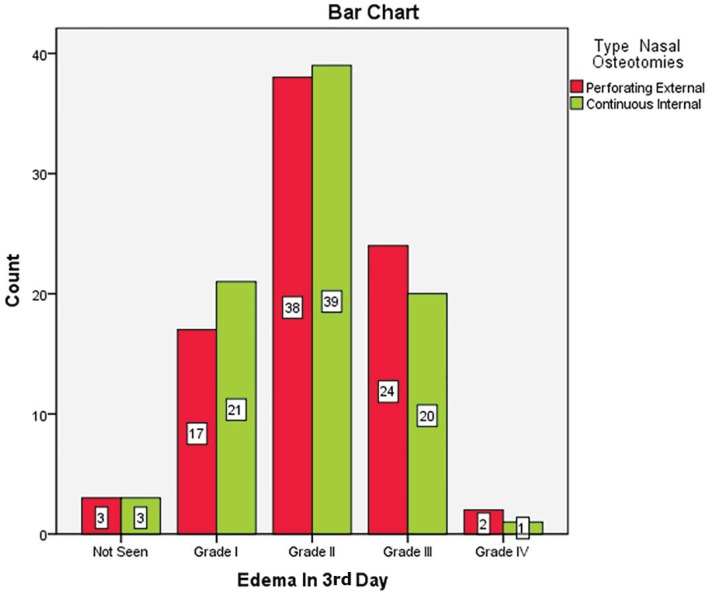
Edema in patients after 3 days of undergoing nasal osteotomy

**Fig. 8:**
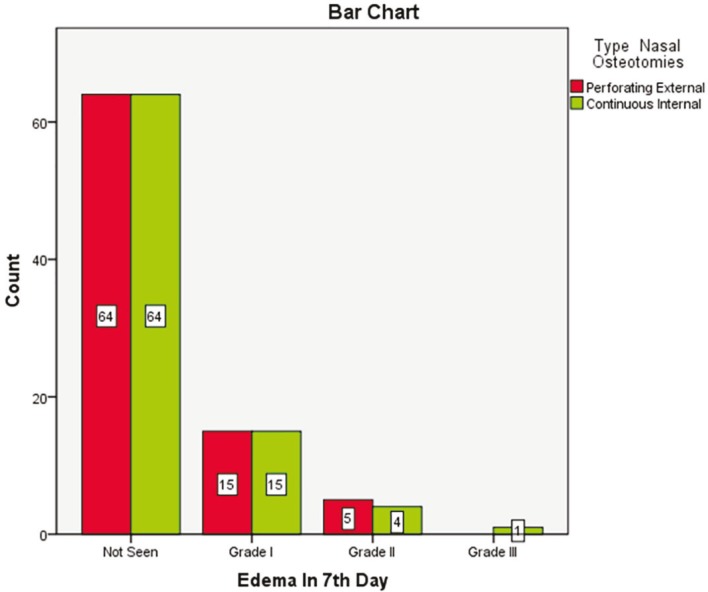
Edema in patients after 7 days of undergoing nasal osteotomy

The most common edema type in the 3^rd^ day was grade 2. Thirty eight patients (45.2%) in external route and 39 cases (46.4%) in internal method had grade 2 edema on the 3^rd^ day and edema rate was not significantly different between the two techniques (*p*=0.9) (Figure 7). On 7^th^ day, edema resolved mostly and 64 patients (76.2%) in external type and 64 cases (76.2%) in internal treatment did not experience any edema and regarding edema, there was not any significant difference between the two groups on 7^th^ day (P=0.9) (Figure 8). In 83 patients (98.8%), edema was resolved on 30^th^ day, but this improvement did not differ between the two routes (P>0.05). On 7^th^ day, in most cases, skin color changed from bluish red to light yellow and there was no difference in routes. 

## DISCUSSION

Nasal bone rearrangement is a vital step in rhinoplasty and past results showed that selection of lateral osteotomy route depends on surgeons` preference not different conclusions of previous studies. It should be reliable, accurate and ideal with fewer sequels. Intranasal soft tissue damage can cause hemorrhage, edema and long-term ecchymosis that affect recovery and rehabilitation. Challenges exist in this era on selecting best option for osteotomy. Our study can be assumed as the first evaluation of osteotomy in rhinoplasty regarding fewer annoying complications, because both types of osteotomy were done in each side of patient`s nose respectively and confounding factors easily were omitted. So, 168 osteotomies were carried on 84 patients (84 internal vs 84 external type). 

Yucel *et al. *(2005) demonstrated that osteotomy types did not differ regarding ecchymosis and edema in favor of our study but with fewer cases.^9^ Van Loon (2011) showed similar swelling after osteotomy using both types of surgeries and confirmed it with 3D streophotogrammetry.^10^ Helal *et al.* (2010) approved the effect of osteotomy on Intra Nasal Valve (INV) without dominancy of any route.^11^ In the other cadaveric study, it was shown that mucosal laceration in internal and external osteotomy were 74% and 11%, respectively.^12^ Giacomarra`s findings also were in agreement with external osteotomy due to its low mucosal damage.^13^

Becker with 30 years’ experience, despite confirming external route`s reliability, suggested internal osteotomy by 2 mm osteotome a without protector to be done without any nasal mucosal damage, though 4 mm guard osteotome had 95% tissue damage.^7^ The findings were confirmed by Patrick and Sullivan too.^14^ Because of dependency to experienced surgeons, we used 4 mm curve osteotome to reduce slip and more tissue damage by ourselves. However, another study with similar instruments like ours (internal osteotomy with 4 mm guard osteotome and external osteotomy with 2 mm osteotome) had less edema and ecchymosis on 2^nd^, 3^rd^ and 7^th^ days in external route.^15^

There are nonsurgical approaches to overcome edema and ecchymosis such as intravenous dexamethasone regime which were not included in the study.^16^ It is expected that ecchymosis would vanish gradually and our study illustrated that it was increased from 1^st^ to 3^rd^ day and then decreased from 3^rd^ to7^th^ day and sometimes was eliminated. There was similarity between two routes as every time, procedures were performed on the same client, most confounding variables were removed automatically and the result was closer to reality.

Most of mentioned studies were retrospective and unilateral, and ours was bilateral and prospective with subjective results.^17^ We provided a complete similar condition for both routes in a patient like Yucel`s study.^9^ There are confounding factors that affect coagulation such as blood pressure, NSAIDs, OCP or other medicines which can be excused in a single patient. Proponents of internal osteotomy cite no escape way for resulted blood in external route and expect more edema and ecchymosis, but lacerated mucosa can drain hemorrhage, by the way angular artery damage is less expected.

On the other hand, some believe in external osteotomy, because it leaves more intact periosteum and diminishes possible narrow nasal space which does not obstruct air flow. Also, there is less lacrimal duct damage in this procedure with resultant catastrophes. As a rule, osteotomy should be selected according to nasal anatomical condition, medical history, surgeon`s experience and easy and safe route. We concluded that osteotomy type did not affect edema and ecchymosis and should follow a general rule.
